# The modern morphometric approach to identify eggs of Triatominae

**DOI:** 10.1186/s13071-017-1982-2

**Published:** 2017-01-31

**Authors:** Soledad Santillán-Guayasamín, Anita G. Villacís, Mario J. Grijalva, Jean-Pierre Dujardin

**Affiliations:** 1 Center for Research on Health in Latin America (CISeAL), School of Biological Sciences, Pontifical Catholic University of Ecuador, Av. 12 de Octubre 1076 y Roca, Quito, Ecuador; 2 Infectious and Tropical Disease Institute, Department of Biomedical Sciences, Heritage College of Osteopathic Medicine, Ohio University, Athens, OH 45701 USA; 3 IRD, UMR 177 IRD-CIRAD INTERTRYP, Campus international de Baillarguet, Montpellier, France

**Keywords:** Chagas disease, Triatominae, Egg, Operculum, Geometric morphometrics, Ecuador

## Abstract

**Background:**

Egg morphometrics in the Triatominae has proved to be informative for distinguishing tribes or genera, and has been based generally on traditional morphometrics. However, more resolution is required, allowing species or even population recognition, because the presence of eggs in the domicile could be related to the species ability to colonize human dwellings, suggesting its importance as a vector.

**Results:**

We explored the resolution of modern morphometric methods to distinguish not only tribes and genera, but also species or geographic populations in some important Triatominae. Four species were considered, representing two tribes and three genera: *Panstrongylus chinai* and *P. howardi*, *Triatoma carrioni* and *Rhodnius ecuadoriensis*. Within *R. ecuadoriensis*, two geographical populations of Ecuador were compared. For these comparisons, we selected the most suitable day of egg development, as well as the possible best position of the egg for data capture. The shape of the eggs in the Triatominae does not offer true anatomical landmarks as the ones used in landmark-based morphometrics, except for the egg cap, especially in eggs with an evident “neck”, such as those of the Rhodniini. To capture the operculum shape variation, we used the landmark- and semilandmark-based method. The results obtained from the metric properties of the operculum were compared with the ones provided by the simple contour of the whole egg, as analyzed by the Elliptic Fourier Analysis. Clear differences could be disclosed between the genera, between the species - among which two very close species (*P. chinai* and *P. howardi*), as well as between two allopatric, conspecific populations. The whole egg contour (including the operculum) produced reclassification scores much more satisfactory than the ones obtained using the operculum only.

**Conclusions:**

We propose the outline-based approach as the most convenient characterization tool to identify unknown eggs at the species or population levels.

**Electronic supplementary material:**

The online version of this article (doi:10.1186/s13071-017-1982-2) contains supplementary material, which is available to authorized users.

## Background

Triatominae are the vectors of *Trypanosoma cruzi*, the causative agent of Chagas disease. Out of the more than 152 species reported in this subfamily, a few are closely adapted to human structures and represent the main vectors. It is admitted however, that all the species are potential vectors, some of them becoming today more important after the elimination of the main vectors during various international campaigns [1–3].

In Ecuador, sixteen species of the Triatominae have been reported, distributed in 20 of the 24 provinces [4, 5]. The main vectors of the country are *Rhodnius ecuadoriensis* and *Triatoma dimidiata*. Other species, belonging to the genera *Triatoma* or *Panstrongylus*, are considered to be secondary vectors [4].

Triatomine eggs are oval or elliptical, slightly asymmetrical and present a smooth convex or ornamented operculum [6, 7]. Eggs have received much attention regarding insecticide sensibility [8–10] or physiology [11, 12]. Systematic studies on the Triatominae were mainly focused on adults and nymphs, and less frequently on eggs [13]. For species [14–22], or population characterization [23–25], egg morphology has been examined mainly on the basis of qualitative characters such as color pattern and structural characteristic (form, texture of shell and operculum, exochorial architecture).

Except for a recent study comparing the chorial rim and the collar of the eggs between various species of *Rhodnius* [19], the morphometric studies always relied on traditional techniques [13, 15, 18, 20, 22–27]. The main problem for the study of elliptical structures (e.g. eggs) through traditional techniques is the difficulty and ambiguity to locate maximum or minimum part of curvature. The modern morphometrics allows a direct description of the contour, separating size and shape, with the additional possibility to visualize shape changes among groups [28].

Frequently, the morphology of the eggs of Triatominae considers separately the operculum and the remaining part of the egg [17, 22, 29]. We thus separated the morphometric analysis of the operculum, performed according to the landmark-based method, and the analysis considering the complete egg (including the operculum), based on the outline-based morphometric approach. For a Triatominae species, the presence of eggs in the domicile could be related to the ability of this species to colonize human dwellings [30], suggesting its importance as a vector. For this reason, it is relevant to develop egg-based species identification techniques. The aims of our study were (i) to apply the modern morphometric techniques for eggs characterization, and (ii) to test these techniques for species and population discrimination.

## Methods

### The insects

**Fig. 1:**
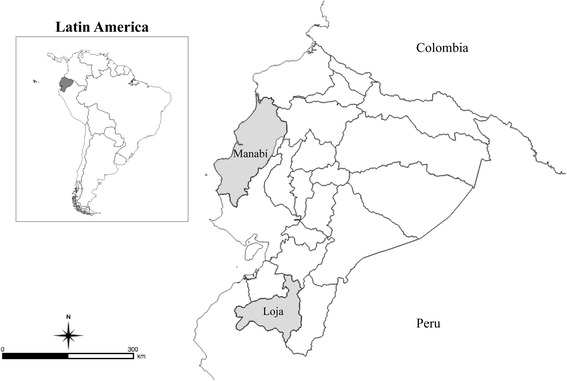
Geographic origin of the specimens in Ecuador. *Panstrongylus howardi* is restricted to Manabí, *P. chinai* and *T. carrioni* are restricted to Loja. *Rhodnius ecuadoriensis* came from both provinces

The geographical limits of our study were two separate provinces in Ecuador: Loja and Manabí (Fig. 1). The Province of Loja, in the southern Andean region of Ecuador, is characterized by a mix of hilly and mountainous topography, with an altitude ranging from 120 to 3800 m above sea level (masl). The region includes inter-Andean temperate valleys and has an average rainfall of 400 mm/year [31]. Manabí Province is located along the central coast of Ecuador at an altitude ranging from 0 to 400 masl, and receives an average annual rainfall of 563 mm/year [31].

*Triatoma carrioni* and *Panstrongylus chinai* were found only in Loja Province, while *P. howardi* was collected from the Manabí Province where it is endemic. Only *Rhodnius ecuadoriensis* was found in both provinces.

The eggs submitted to morphometric analyses were obtained from field-collected females having spent various generations in the laboratory, except for *T. carrioni* for which eggs were obtained from the field. We separated individuals of *Panstrongylus* and *Rhodnius* during the fifth-instar stage, and established colonies with virgin females. The colonies were maintained in the insectary of the Center for Research on Health in Latin America (CISeAL), Pontificia Universidad Católica del Ecuador (PUCE) under controlled conditions of 25 ± 6 °C, 70 ± 5% relative humidity (RH) for Loja specimens, 27 ± 5 °C, 75 ± 5% RH for Manabí specimens, and a photoperiod of 12:12 h (Light:Dark) for specimens from both provinces. Blood meals were offered each 15 days during 30 min, using immobilized pigeon (*Columba livia*).

The 78 *P. chinai* eggs came from 48 females (6 localities), the 75 *P. howardi* eggs came from 8 females (one locality), and the *T. carrioni* eggs came from 7 females (4 localities). The 73 and 76 *R. ecuadoriensis* eggs came from 48 females (5 localities) in Loja and 24 females (3 localities) in Manabí, respectively (Table 1).

**Table 1:** Geographical and year origin of parents used for obtaining the eggs

Species	Province	Year	*N* ^a^
*R. ecuadoriensis*	Manabí	2005–2013	73
	Loja	2005–2013	76
*T. carrioni*	Loja	2015	76
*P. chinai*	Loja	2006–2011	78
*P. howardi*	Manabí	2007	75
Total			378

*Abbreviations*: *R Rhodnius*, *T Triatoma*, *P Panstrongylus*

^a^N, number of eggs analyzed by species

### Material preparation and photography

**Fig. 2:**
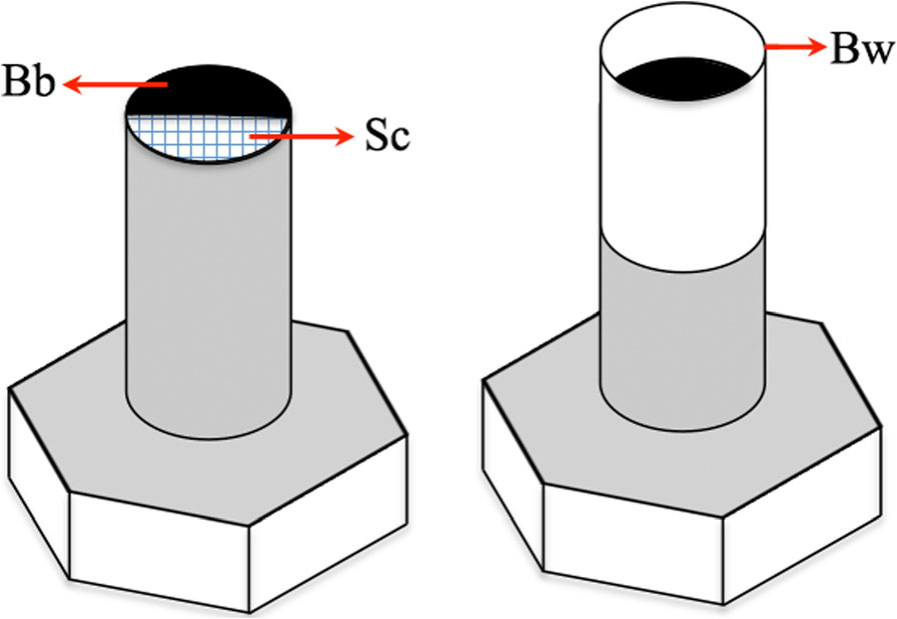
Platform device for the eggs. The platform was a bolt with, on top, a black paper (background) and a semicircular graph paper (scale), protected by a wall for biosecurity. *Abbreviations*: Bb, black background; Sc, scale; Bw, biosecurity walls

To ensure a reproducible protocol of image capture from one individual to another, eggs were deposited on a small platform as described Fig. 2 and photographed using a MiScope-MIP (www.zarbeco.com). On the picture, the scale was visible and used to convert the coordinates from pixels to millimeters. Using this device, we checked the possible effect of position and/or developmental stage on size and shape (see below).

#### Egg position

We tried to choose the same position for all individuals; the possible problem induced by the position of the egg was treated separately for “neck-less” eggs (Triatomini) and the others (Rhodniini).

**Fig. 3:**
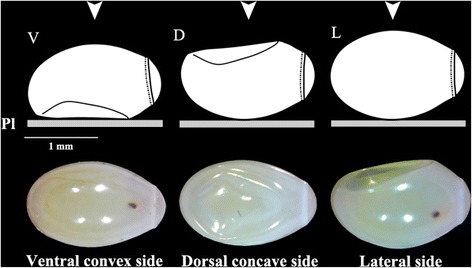
*Panstrongylus chinai* egg in ventral (left), dorsal (center) and lateral (right) positions. The local flattening is located on the dorsal side of the egg. The drawings at the top of the figure show the eggs as deposited on the platform (Pl, see Fig. 2), the three bottom pictures are the eggs as envisioned by the camera objective (see the vertical arrow on the top). According to the egg position on the platform, the camera captures a possible different contour, which is obvious when the egg is put on its lateral side (rightmost picture). *Abbreviations*: Pl, platform; V, ventral side; D, dorsal side; L, lateral side

The neck-less eggs studied for possible position interference on shape were those of *P. chinai*. Ten days after oviposition, the eggs of *P. chinai* present a ventral convex side and dorsal concave side caused by the appearance of a local flattening (Fig. 3). When the egg is deposited on its dorsal side, the egg lies stable on its flattened part, the camera is viewing the ventral side of the embryo, so that the flattened part is not visible. When the egg is deposited on its ventral side, the camera is viewing the dorsal face of the embryo; the flattened part is visible although apparently not affecting the shape of the external contour (Fig. 3). In the lateral position of the embryo, i.e. in the position where the lateral spots of segments of the embryo are visible, the external contour as seen by the camera is truncated at the location of the flattened part. We compared the size and the shape of eggs contours between ventral (*n* = 13) and dorsal (*n* = 13) positions (Fig. 3).

In *R. ecuadoriensis*, the lateral position was the only position that allowed to distinguish the neck of the egg. To have the possibility to examine the neck, the lateral position was thus selected for our study. Thus, the Triatomini were photographed in ventral position while the Rhodniini were photographed in lateral position.

#### Egg developmental stage

**Fig. 4:**
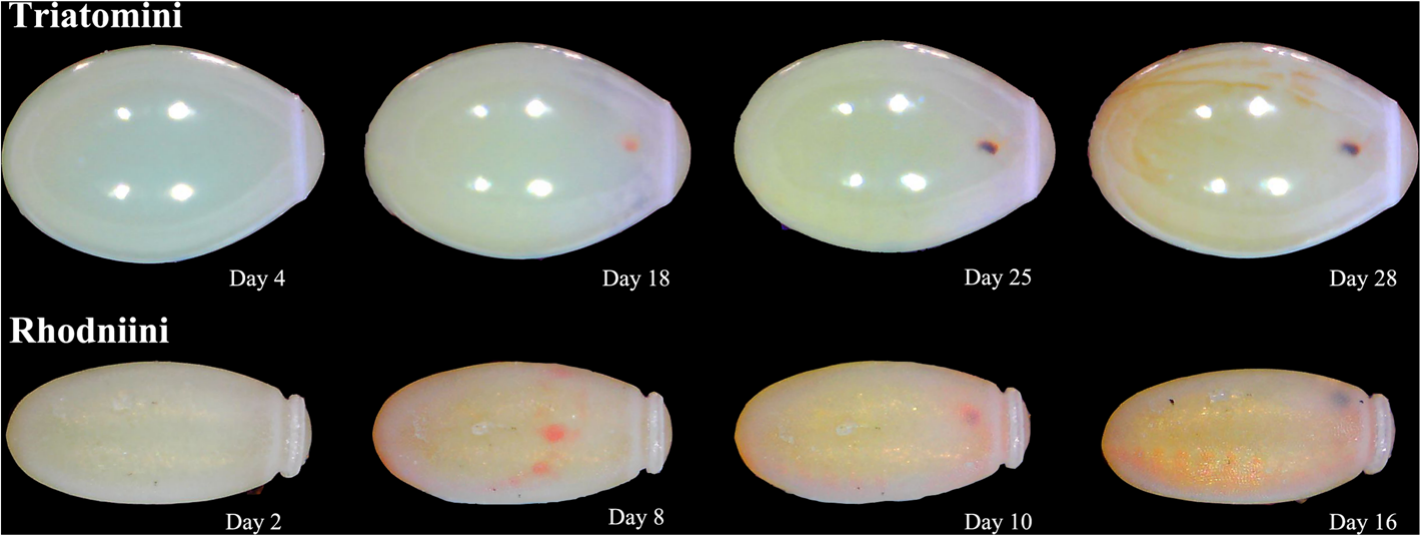
Eggs of *Panstrongylus chinai* and *Rhodnius ecuadoriensis* (Loja) in different development days. The eggs without visible eye-spots or with these spots at the posterior position were considered as “early stage” eggs (less than 14 days in Triatomini or less than 9 days in Rhodniini). The eggs with visible eye-spots at their anterior part (operculum area) were labelled “last stage” eggs (more than 21 days in Triatomini or more than 14 days in Rhodniini). In this picture, the egg of *R. ecuadoriensis* is lying on its ventral, convex side

The 378 eggs of the present study were viable eggs. For *P. chinai*, size and shape of the egg contour were compared between the first 14 days (*n* = 36) and the days 21 to 28 (*n* = 24) (Fig. 4). These features were compared between the first nine days (*n* = 33) and the days 14 to 18 (*n* = 22) for *R. ecuadoriensis* (Fig. 4). These comparisons were performed on eggs maintained in the same position/orientation by gluing them on a piece of paper since the first day.

We tried to choose individuals of the same development stage, but the development time was different between genera (*P. chinai*: 29.23 ± 1.521 days, *P. howardi*: 29 ± 1.296 days, *T. carrioni*: 25 ± 0.787 days and *R. ecuadoriensis*: 17 ± 1.457 days). *Panstrongylus* eggs were photographed in the day 25 of development, *Triatoma* eggs in the day 20–23 of development and *Rhodnius ecuadoriensis* eggs in day 10 of development. An additional criterion for the number of days was the presence of visible, darker eye-spots in the operculum zone (anterior part of the egg). This criterion was used for selecting the *T. carrioni* eggs, which were obtained from field-collected females.

### Statistical approaches

We applied two different geometric approaches according to the anatomical part which was considered, either the complete contour of the egg (outline-based morphometrics) or the operculum (landmark/semilandmark-based morphometrics). Both landmark/semilandmark- and outline-based approaches included two steps: (i) extraction of size and shape variables, which is specific to the technique used, and (ii) discrimination using final shape variables.

Measurement error was examined by the repeatability index [32]. Thus, for each genus (*Panstrongylus*, *Triatoma* and *Rhodnius*), we used an ANOVA design on repeated measurements performed on a sample of 30 eggs.

#### Egg contour (outline-based approach)

**Fig. 5:**
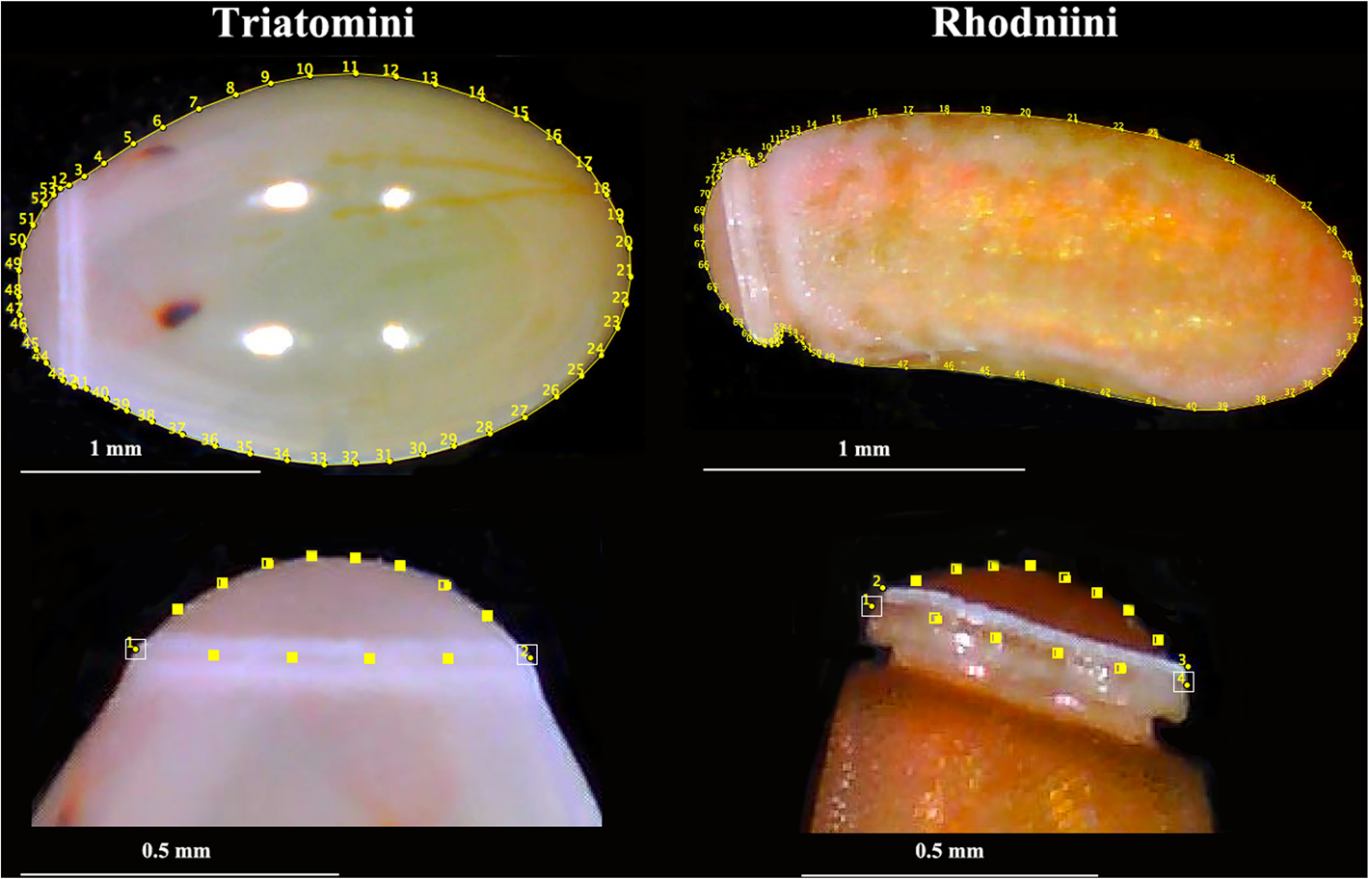
Outline as digitized for the eggs of Triatomini (left side) and Rhodniini (right side). For each tribe, the contour is presented in the upper part of the figure and the operculum in the bottom part. For the contour of the egg, numbered points are pseudolandmarks. For the operculum, points with a number are landmarks, others are semilandmarks. For the Rhodniini, four landmarks were possible, while only two landmarks were possible for the Triatomini

The outline considered was simply the external contour of the egg (Fig. 5). Size was estimated as the square root of the internal area of the contour, as well as the perimeter of the contour; these were compared between groups using non-parametric tests. For shape variable definition, we exclusively used the elliptic Fourier analysis (EFA) [33]. Briefly, the observed contour is decomposed in terms of sine and cosine curves of successive frequencies called harmonics, and each harmonic is described by four coefficients. With this method, the first harmonic ellipse parameters are used to normalize the elliptic Fourier (NEF) coefficients so that they are invariant to size, rotation, and the starting position of the outline trace. By doing this, the three first coefficients become constant (1, 0 and 0) and are not used in the remaining analyses. The fourth coefficient, the one related to the width-to-length ratio of the outline, has been used in our study. The EFA algorithm does not require the points to be equidistant, nor does it require them to be in the same number [34].

To accurately represent a closed curve, many harmonics may be needed, each one with four coefficients, so that the number of variables could be too numerous relative to the number of individuals. The normalized coefficients (NEF) were thus submitted to a principal components analysis (PCA), and the principal components (PCs) were the final shape variables. This procedure allows for reducing the number of input variables (PC) where necessary. Statistical comparisons of size and shape between species were based on non-parametric, permutation-based tests (1,000 cycles) after Bonferroni correction.

#### Operculum shape (semilandmark-based approach)

In *Rhodnius*, 4 unambiguous landmarks could be used, while 2 landmarks only could be used in *Triatoma* and *Panstrongylus* (Fig. 5). Since it was not possible to ascertain the anatomical homology between tribes, the operculum was analyzed based on a different number of landmarks according to the presence (Rhodniini) or not (Triatomini) of a neck. In addition to the landmarks selected on the operculum, 8 semilandmarks were used to capture the external curved line of the operculum (Fig. 5) and 4 semilandmarks were used to trace the curved limit between the egg and operculum.

All landmarks and semilandmarks were submitted to partial Procrustes superimposition [35] and semilandmarks then subjected to a sliding procedure [36, 37]. The tangent space projections [38] were used as input for a principal components analysis, and these principal components were retained as final shape variables. The centroid size was estimated as the square root of the sum of the squared distances between the center of the configuration of landmarks and each individual landmark [39].

#### Shape-based discrimination

For the egg contour, each pairwise Mahalanobis distance was computed between the principal components of the normalized elliptic Fourier coefficients. For the operculum, the distance was computed between the PC of the tangent space variables. The Mahalanobis distances were used to test for validated reclassification, as well as for building a Neighbor-Joining tree (NJ).

#### Contribution of size to shape discrimination

Contribution of size variation to shape-based discrimination was estimated through the determination coefficient between the first discriminant factor and the estimator of size. The determination coefficient was compared between tribes, genera, species and geographic conspecific populations.

### Software

Digitization and statistical methods (EFA, Procrustes superposition, multivariate analyses) used the *CLIC* package (http://mome-clic.com). Semilandmarks slicing was repeated using the “*geomorph*” R package [40] and compared with *CLIC* results. Neighbor-Joining tree (NJ) was constructed using the “*ape*” R package [41].

## Results

Measurement error was very low, below 3% for the egg contour estimation (*P. chinai*, *T. carrioni* and *R. ecuadoriensis*, with repeatability indices of 0.97, 0.98 and 0.98, respectively); the measurement error was slightly higher for the operculum (repeatability indices of 0.95, 0.97 and 0.99, respectively). The measurement error for size was even lower (< 1% for mean square root of the area and perimeter, and < 4% for centroid size).

### Comparison between egg positions

The comparison of ventral and dorsal positions of *P. chinai* eggs did not reveal detectable difference in shape (permutation test, 1000 cycles, *P* = 0.610), nor did it in size (permutation test, 1000 cycles, *P* = 0.732 and *P* = 0.758 for square root area and perimeter, respectively) (Fig. 3).

### Comparisons between egg developmental stages

The size of different egg stages for *P. chinai* did not change significantly, be it estimated by the square root area or by the perimeter. For *R. ecuadoriensis*, the square root area - but not the perimeter - showed a highly significant reduction (permutation test, 1000 cycles, *P* < 0.0001) in the late developmental stage. The same trend was observed for *P. chinai*, although not statistically significant (permutation test, 1000 cycles, *P* = 0.098 and *P* = 0.271 for square root area and perimeter, respectively).

**Fig. 6:**
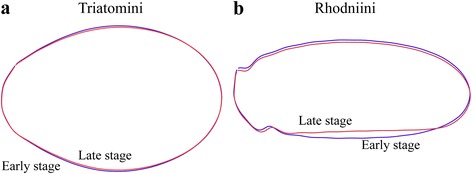
Shape differences between outlines of early and late stage of eggs. **a** Egg of *P. chinai* (Triatomini). **b** Egg of *R. ecuadoriensis* (Rhodniini). The shape of late stage tends to be more slender

Shape differences between early and late stage were found significant for both species (permutation test, 1000 cycles, *P* = 0.009 and *P* < 0.0001 for *P. chinai* and *R. ecuadoriensis*, respectively): the late stage eggs tended to be more slender (Fig. 6). In this shape variation, size contributed unequally according to the species, 18.8% for *P. chinai* and 4.6% for *R. ecuadoriensis*.

### Comparisons of size between groups

The species with smallest eggs was *R. ecuadoriensis. Panstrongylus chinai* contained larger eggs than *P. howardi*, in spite of presenting a significantly smaller operculum (Table 2). The eggs of *Rhodnius* and of *Panstrongylus* whose parentals were collected in Manabí presented a significantly larger operculum (Table 2).

**Table 2:** Size variation of the complete egg (sqrA and Perimeter) and of the operculum (Centroid size)

Species		sqrA		Perimeter		Centroid size	
		Mean	Variance	Mean	Variance	Mean	Variance
*R. ecuadoriensis*	L	1.07	0.0019	4.51^c^	0.038	0.67	0.0012
	M	1.09	0.002	4.65^b^	0.037	0.74	0.0012
*T. carrioni*		1.22	0.0029	4.64^b^	0.042	0.68	0.0013
*P. chinai*		1.32	0.0025	4.91^a^	0.031	0.72	0.0018
*P. howardi*		1.28	0.0025	4.71^b^	0.035	0.78	0.002

*Abbreviations*: *R Rhodnius*, *L* Loja Province, *M* Manabí Province, *T Triatoma*, *P Panstrongylus*, sqrA, mean square root of the area within the egg boundary

*Note*: All pairwise comparisons of mean sqrA were significant (permutation test, 1000 cycles, *P* ranging from *P* = 0.015 to *P* < 0.0001). The perimeter showed significant differences between three groups: (a) *P. chinai*, (b) *P. howardi*, *T. carrioni* and *R. ecuadoriensis* Manabí and (c) *R. ecuadoriensis* Loja (permutation test, 1000 cycles, *P* < 0.0001). The only significant difference of variance was observed for sqrA between *T. carrioni* and the Loja sample of *R. ecuadoriensis* (permutation test, 1000 cycles, *P* = 0.002). The mean size of the operculum was significantly different between all comparisons (permutation test, 1000 cycles, *P* < 0.0001). The variance of the operculum size was not significantly different between groups

The mean square root of the area (sqrA) within the egg boundary was significantly different in all pairwise comparisons (Table 2). The perimeter variation was less resolutive, except for the comparisons between three groups: (a) *P. chinai*, (b) *P. howardi*, *T. carrioni* and *R. ecuadoriensis* Manabí and (c) *R. ecuadoriensis* Loja.

For the operculum variance of size, or for the variance of the perimeter of the egg, no significant trend could be disclosed between groups. However, the variance of sqrA showed significant difference between *T. carrion*i and one sample of the *R. ecuadoriensis* (Table 2).

### Comparisons of shape between groups

**Fig. 7:**
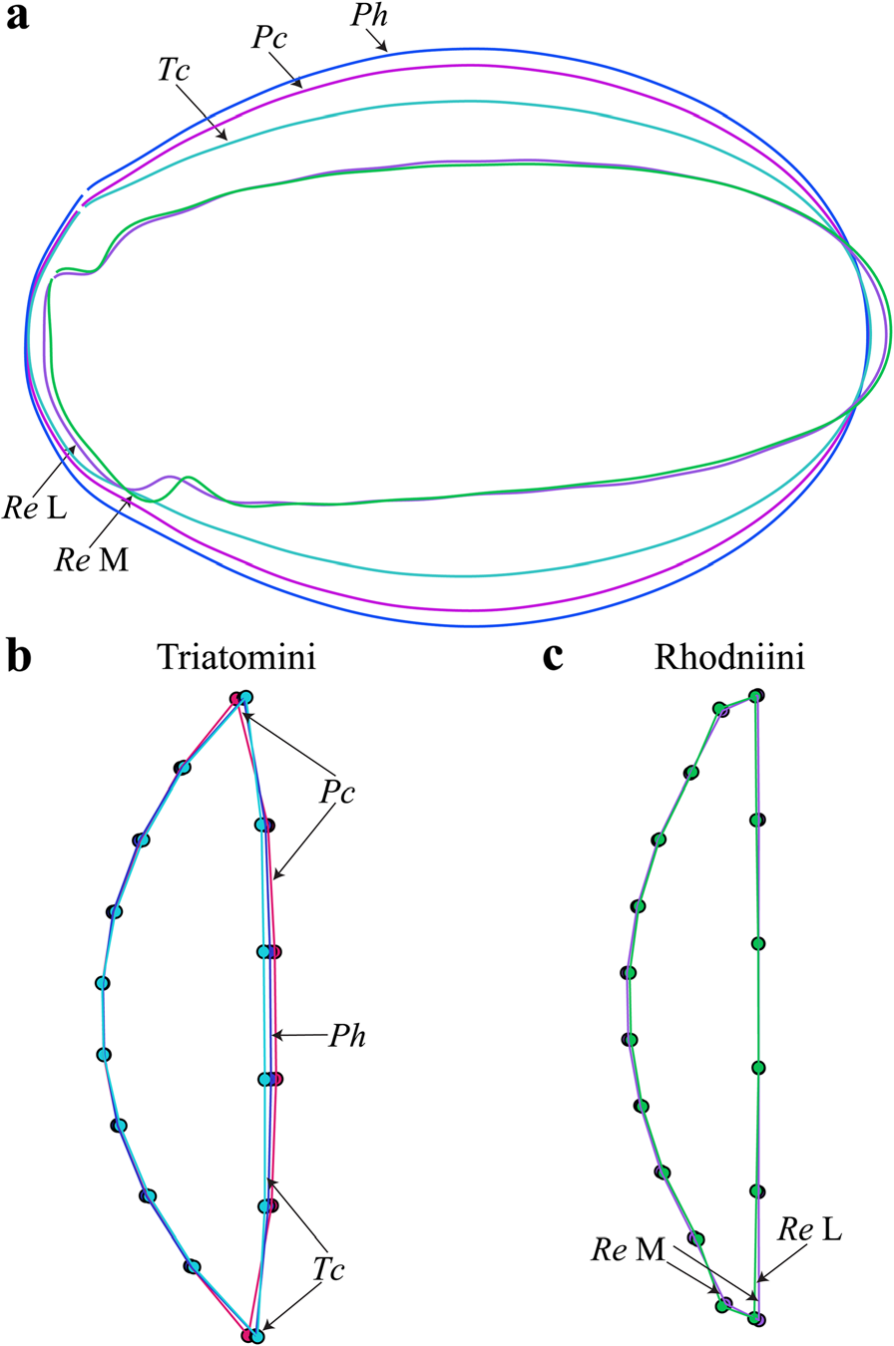
Egg and operculum shape. **a** Egg shape differences between outlines of *P. chinai*, *P. howardi*, *T. carrioni* and two populations of *R. ecuadoriensis* (Loja and Manabí). Differences in egg shape between species and populations were visible without special amplification. **b** Differences in operculum shape between *P. chinai*, *P. howardi* and *T. carrioni*. **c** Differences in operculum shape between the two populations of *R. ecuadoriensis*, Loja and Manabí. *Abbreviations*: *Pc*, *Pantrongylus chinai*; *Ph*, *P. howardi*; *Tc*, *Triatoma carrioni*; *Re* L, *Rhodnius ecuadoriensis* Loja; *Re* M, *R. ecuadoriensis* Manabí

All pairwise comparisons of shape were highly significant (permutation test, 1000 cycles, *P* < 0.0001), for either the egg contour or the operculum shape comparisons. Differences in egg shape between species and populations were visible without image amplification (Fig. 7). Among “neck-less” eggs (Triatomini), *T. carrioni* was the most slender and, *P. howardi* the widest. The main difference among populations of *R. ecuadoriensis* was located at the neck size and orientation (Fig. 7).

The mean Mahalanobis distance based on the egg shape was 26 between tribes, 6.3 between species and 6.5 between the geographical populations of *R. ecuadoriensis*. The Mahalanobis distance based on the operculum shapes was on average 1.8 between the Triatomini species and 2.4 between the two *R. ecuadoriensis* populations.

Reclassification scores (Table 3) among species were excellent when obtained from the contour of the whole egg (93.2%, on average), but less satisfactory when based on the operculum shape (67.7%, on average). Between the two geographical populations of *R. ecuadoriensis*, the reclassification score based on the operculum was relatively high (81.2%), but lower than that based on the egg contour (90.6%).

**Table 3:** Correct reclassification scores for the complete contour of the egg and for the operculum

Species		Egg		Operculum	
		*N * ^a^	%^b^	*N* ^a^	%^b^
*R. ecuadoriensis*	M	63/73	86	60/73	82
	L	74/76	97	61/76	80
*T. carrioni*		74/76	97	55/76	72
*P. chinai*		71/78	91	49/78	62
*P. howardi*		70/75	93	51/75	68

*Abbreviations*: *R Rhodnius*, *L* Loja Province, *M* Manabí Province, *T Triatoma*, *P Panstrongylus*

^a^N, number of correctly assigned individuals/Total of individuals analyzed by group

^b^%, percent of correctly assigned individual

**Fig. 8:**
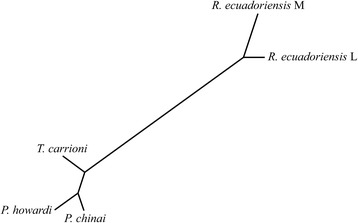
Neighbor-joining trees derived from egg shape variation based on Mahalanobis distances for egg shape between *P. chinai*, *P. howardi*, *T. carrioni* and two populations of *R. ecuadoriensis* (Loja and Manabí). *Abbreviations*: *P*, *Panstrongylus*; *T*, *Triatoma*; *R*, *Rhodnius*; L, Loja; M, Manabí

The Neighbor-Joining tree (NJ) based on the external contour of the eggs revealed (i) remarkable difference between Triatomini and Rhodniini eggs; (ii) correct clustering of three species belonging to two genera, *Panstrongylus* and *Triatoma*; and (iii) distinction between two geographic populations within *R. ecuadoriensis* (Fig. 8).

### Contribution of size to shape discrimination

The influence of size variation on shape distinction was examined in the comparison between tribes (74%), between the genera *Panstrongylus* and *Triatoma* (egg 15.8%, operculum 6%), between the two *Panstrongylus* species (egg 13.3%, operculum 26.7%), as well as between the *Rhodnius* subpopulations (egg 4%, operculum 41.4%).

## Discussion

For metric characters in general, there are many possible causes for size or shape variation between organisms [42]. According to the literature available on insect eggs, one possible reason for shape changes could result from an artefact related to the position of the egg, or could be the effect of egg developmental age, or to some other parameters related to the mother and/or the environment.

The choice of the same stage of development for species comparisons was justified because the egg physiology suggests possible change of size during development. For instance a possible shrinking of the egg volume could be expected because of yolk lipids consumption by the embryo, as shown in *R. prolixus* [43, 44]. Chaves et al. [12] identified an increase of the maximum diameter of eggs during development, but Hinton [45] and Beament [46] showed that the size of the egg was reduced because of water loss. In their study, Chaves et al. [12] suspected the eggs could have been mishandled so that the final diameter increase could be spurious data. We discarded this possible source of artefact by gluing the eggs in the same position when examining the possible changes during development. The difference of shape we found between development stages could appear as a difficulty for studying eggs since it means that eggs should be selected for their age before comparisons. However, the simple criterion of visible eye-spots in the anterior part of the egg (Fig. 4), as used here for *T. carrioni*, is valid. To avoid possible data heterogeneity, we recommend however to try comparing eggs at a similar developmental stage.

The physiological status of females could influence also some traits of their eggs [11, 47], a possible effect which was taken in account here by comparing eggs coming from newly-molted females. By comparing laboratory lines reared under the same conditions, fed with the same source of blood at the same frequency, using eggs at similar developmental stages and positions, we could reduce the possible environmental influence on egg shape variation. The clear-cut differences found between some groups, especially between species, are thus unlikely to be artefactual or circumstantial differences.

*Panstrongylus chinai* and *P. howardi* are controversial species [48]. However, they were almost perfectly separated (96%) by the shape of the egg, and weakly (74%) separated by the shape of the operculum. As an additional argument for diverging forms, they showed no parallelism between the respective sizes of the egg body and the operculum. This level of divergence was in contrast with a recent morphometric study of the head and wings showing relatively low morphometric and cytogenetic differences, compatible with geographical variation (Additional file 1: Table S1). The difference of egg shape could perhaps be attributed to the restricted spatial and temporal sampling of *P. howardi*: their eggs were collected from a single community and on the same day. As a consequence, our *P. howardi* sample could provide a truncated representativeness of the species variability. The other reason for the clear-cut difference as disclosed between *P. howardi* and *P. chinai* could be, however, true evolutionary difference.

In addition to species discrimination, the egg shape disclosed consistent differences between allopatric populations of *R. ecuadoriensis*. This is in agreement with the variation observed in the geometry of their wings, suggesting some genetic drift due to geographical isolation, not excluding environmental effects due, for instance, to different hosts or different climates [49]. The level of differences between these two geographic populations was striking: the Mahalanobis distances as based on the egg contour (6.5) were larger than between species of the Triatomini (6.3), and larger also (2.4 *versus* 1.8) when based on the operculum. Accordingly, the reclassification scores were comparable (egg contour) as, or higher (operculum) than, the ones obtained between species of the Triatomini. Moreover, in spite of living in a warmer climate and at a lower altitude, the Manabí specimens tended to be larger, which contradicts the Bergmann’s rule and could suggest genetic divergence [50]. However, the Bergmann’s rule argument is difficult to consider between populations located close to the equatorial line [51].

## Conclusions

The geometric morphometrics applied to the eggs - contrary to the one applied to the wing venation of insects - is still in its infancy, and, due to its promising features, more studies are expected. An obstacle could be the difficulty to find eggs in the field because of the size of the eggs, the inaccessible places of oviposition (cracks of walls) and the possibility to find only the chorion (without operculum). This problem is true for most Triatomini eggs, but can be ruled out for the gluing eggs such as those of Rhodniini: the *R. ecuadoriensis* eggs were found fastened in abundance to various substrates, like chicken nests, clothes, walls, or any material accumulated in the domicile and peridomicile.

Because the eggs presented higher discrimination power than heads or wings, as between *P. chinai* and *P. howardi*, the method developed here should be recommended. Another reason for such recommendation could be related to the digitization facilities when dealing with a simple egg shape. Such form is liable to be digitized automatically (see for instance the Conte function in [52]), and an automatic digitization could speed up the data collection and reduce the measurement error. But even after manual digitization, the measurement error was very low (2.33% for the outline-based approach, 5% for the semilandmarks-based one).

Our data open new avenues of research. Due to the promising virtues of egg morphometrics, more species comparisons may be expected, and from an epidemiological point of view, it would be important to check for possible discrimination between eggs whose parentals were collected in different habitats (domicile, peridomicile and sylvatic) or between hatched eggs (eggs without operculum). Eggs should not be used only for systematic purposes: we could expect indeed their shape to witness some changes due to impaired viability, or to natural hybridisation as found between many “species” in the Triatominae [53]. Moreover, their symmetry (“object symmetry”, as described by Mardia et al. [54]) could help exploring the external or internal sources of developmental stress. Finally, some technical improvements should be considered in the near future, especially the testing of automatic digitization.

## Additional files

Additional file 1:
**Table S1.** Reclassification score of head and wing of *P. chinai* and *P. howardi*. The sample sizes for heads and wings were the same, 18 *P. chinai versus* 15 *P. howardi* in females, and 25 *P. chinai versus* 21 *P. howardi* in males. *P. chinai* was collected in the Loja province of Ecuador, *P. howardi* is endemic to the Manabí province of Ecuador. (PDF 70 kb)
Additional file 2: Database Coordinates of the complete contour of the eggs (outline-based method) and of the operculum (landmark/semilandmark-based approach). (XLSX 834 kb)

## Abbreviations

EFA: Elliptic fourier analysis
NEF: Normalized elliptic fourier
NJ: Neighbor-joining
PC: Principal component
PCA: Principal components analysis.
