# Supplementary material for: The modern morphometric approach to identify eggs of Triatominae
Source: Parasit Vectors. 2017 Jan 31;10:55. doi: 10.1186/s13071-017-1982-2 (PMC5286694; doi:10.1186/s13071-017-1982-2)
Supplement: Additional file 1: — Table S1. Reclassification score of head and wing of P. chinai and P. howardi. The sample sizes for heads and wings were the same, 18 P. chinai versus 15 P. howardi in females, and 25 P. chinai versus 21 P. howardi in males. P. chinai was collected in the Loja province of Ecuador, P. howardi is endemic to the Manabí province of Ecuador. (PDF 70 kb) [file 13071_2017_1982_MOESM1_ESM.pdf]

Additional file 1. Table S1. Reclassification score of head and wing of *Panstrongylus chinai* and *Panstrongylus howardi*.

Species	Head Female	Male	Wings Female	Male
P. chinai	77%	80%	66%	92%
P. howardi	73%	76%	93%	85%

P, *Panstrongylus*

The sample sizes for heads and wings were the same, 18 *P. chinai* versus 15 *P. howardi* in females, and *25 P. chinai* versus *21 P. howardi* in males. *P. chinai* was collected in the Loja province of Ecuador, *P. howardi* is endemic to the Manabí province of Ecuador.
